# Epidemiologic Investigation of a Varicella Outbreak in an Elementary School in Gyeonggi Province, Republic of Korea

**DOI:** 10.3390/children12070949

**Published:** 2025-07-18

**Authors:** Gipyo Sung, Jieun Jang, Kwan Lee

**Affiliations:** 1 Pyeongtaek-si Songtan Public Health Center, Pyeongtaek 17730, Republic of Korea; z1z1x1@korea.kr; 2 Department of Preventive Medicine, College of Medicine, Dongguk University, Gyeongju 38067, Republic of Korea; jieunjang@dongguk.ac.kr

**Keywords:** varicella, chickenpox, epidemiologic investigation, outbreak, vaccine-preventable disease, school health

## Abstract

**Background/Objectives**: On 6 June 2023, two varicella cases were reported at a highly vaccinated elementary school in Gyeonggi Province, Republic of Korea. We investigated the outbreak to describe its transmission dynamics; quantify attack rates in school, household, and private-academy settings; and assess the impact of coordinated control measures. **Methods**: A case-series study included 89 teachers and students who had contact with suspected patients. Using case definitions, laboratory tests, questionnaires, and environmental assessments, we evaluated exposures and factors facilitating spread. **Results**: Varicella developed in 23 of 89 contacts (25.8%); laboratory confirmation was obtained in 2 (8.7% of cases). The mean incubation period was 13 days. Epidemic-curve and network analyses indicated that the outbreak began with a single index case and extended through household contacts and private educational facilities, ultimately involving multiple schools. **Conclusions**: Breakthrough transmission can occur even when single-dose coverage exceeds 95%, particularly as vaccine-induced immunity may wane over time. Poorly regulated extracurricular facilities, such as private academies, act as bridging hubs that amplify spread across grades and even between schools. For timely detection and control, these venues should be incorporated into routine varicella surveillance, and rapid, coordinated infection-control measures are required across all educational settings.

## 1. Introduction

The varicella zoster virus (*Human alphaherpesvirus 3*) causes both varicella (chickenpox) through primary infection and herpes zoster (shingles) through endogenous reactivation during latency. Varicella occurs worldwide; in temperate countries, the age of infection tends to be lower than that in tropical countries. In countries without vaccination programs, over 90% of adolescents are infected. Varicella virus is prevalent during winter and spring or in cooler, drier months, and large-scale outbreaks can occur every 2–5 years. It is highly contagious, with a secondary attack rate of 61–100% [1]. Even among vaccinated individuals, 11–17%—and up to 40%, in some instances—may still develop varicella [2]. Transmission occurs primarily through aerosol inhalation from the vesicular fluid of skin lesions, direct contact with rashes, or infected respiratory secretions. Without vaccination, almost the entire population becomes naturally infected with varicella by adulthood [1].

The main symptoms of varicella infection include fever, malaise, and rashes. Notably, the rash consisted of new skin lesions that progressed from the macules to papules, vesicles, and crusts over a period of 5–7 days. Varicella can rarely cause severe complications such as pneumonia, cerebellar ataxia, and encephalitis. The incubation period was generally 14–16 days, with an average of 10–21 days. Transmission can occur 1–2 days before the onset of the rash, until the lesions crust over [1].

Policies on varicella vaccination differ markedly worldwide [3,4]. High-income countries such as Germany, Greece, Italy, and the United States fund a routine two-dose schedule through national programs [3,4], whereas other affluent nations, including Belgium and Austria, require individuals to cover the full cost [4,5,6]. Notably, the Republic of Korea still recommends only a single varicella dose, a policy that has been associated with lower long-term effectiveness compared with the two-dose schedules adopted in the United States, Germany, and Greece [3,7,8]. Many low- and middle-income countries have yet to introduce the vaccine at all [9]. Although the exact global incidence is difficult to estimate, the World Health Organization attributes approximately 4.2 million severe cases and 4200 deaths to varicella each year [10], underscoring the substantial clinical and economic burden of the disease and the need for optimized vaccination strategies. A recent meta-analysis that included 78 studies showed a pooled incidence of severe varicella of 22.4% (95% CI, 10.1–37.8) across 12 hospital-based investigations among admitted patients [11]. These data indicate that breakthrough infections continue to impose a clinical burden even in countries with high vaccination coverage, such as the United States and Türkiye, thereby underscoring the need for rigorous outbreak investigations.

In the Republic of Korea, the National Immunization Program has recommended a single varicella vaccine dose at 12–15 months of age since 2005 [2,12]. Annual case counts reached 80,000–90,000 from 2017 to 2019 but declined to 31,430 in 2020, 20,929 in 2021, and 18,547 in 2022 during the coronavirus disease 2019 (COVID-19) pandemic; nevertheless, 18,008 cases had already been reported by epidemiologic week 38 of 2023 [13,14]. The national varicella vaccination rate was 97.7% for children aged 2 years in 2021 [15], and since July 2005, varicella has been managed under a comprehensive surveillance system as a legally designated communicable disease.

We conducted an epidemiological investigation to identify the scope and causes of a varicella outbreak among elementary school students in a city in Gyeonggi Province during the comprehensive monitoring and management of hospital-reported cases using the Integrated Disease and Health Management System. By identifying the presumed source of infection for the outbreak, we aimed to minimize the scale of infection in similar future outbreaks.

Between 2016 and 2019, 59.6–64.8% of reported varicella outbreaks in Korea occurred in elementary schools, with annual attack rates ranging from 2.5% to 6.9% [16]. Comparable breakthrough outbreaks have been documented in highly vaccinated populations in the United States, Türkiye, and other countries [17,18].

Despite these observations, the role of private after-school institutes in sustaining varicella transmission has not been systematically studied in Korea. Therefore, this study aimed to fill that gap by integrating school-, household-, and academy (hagwon—fee-based tutoring institutes that operate outside regular school hours in Korea)-level data from a single outbreak.

## 2. Materials and Methods

### 2.1. Outbreak Notification

During the comprehensive monitoring and management of facility-reported cases through the Integrated Disease and Health Management System, two individuals clinically suspected of having varicella were reported on 6 June 2023 among fifth-grade students in Class 6 at Elementary School A, Gyeonggi Province (rash onset: 5 June 2023). Both index patients had received a single (first-dose) varicella vaccine, had no underlying medical conditions, came from middle-income households, and attended at least one private after-school academy. Because two or more cases occurred within the same group during a 3-week period, the cluster was classified as an outbreak, prompting initiation of an epidemiologic investigation.

### 2.2. Case Definitions

According to the guidelines for the management of vaccine-preventable diseases issued by the Korea Disease Control and Prevention Agency (KDCA) [2,19], patients were defined as individuals who, from 16 May to 3 September 2023, had contact with individuals suspected of having varicella at the Elementary School A, exhibited clinical symptoms consistent with varicella (e.g., rash, fever, and headache), and were diagnosed with suspected varicella infection at medical facilities or met the diagnostic criteria for varicella. Individuals diagnosed with varicella virus-related meningitis or encephalitis and confirmed positive by testing without exhibiting clinical symptoms of disseminated herpes zoster or varicella were excluded.

The observation period (16 May–3 September 2023) was defined, in accordance with the 2023 KDCA guidelines for vaccine-preventable diseases, as the period extending from 21 days before the first clinically suspected case (6 June 2023) to six weeks after the last identified case (24 July 2023). This interval equals twice the guideline-specified maximum incubation period of varicella and was chosen to ensure that every case linked to the outbreak was captured. In contrast, because complete attendance rosters from the academies were unavailable, only passive surveillance could be implemented.

### 2.3. Study Design

A case-series study was conducted involving 81 students and 4 teachers from Elementary School A in Gyeonggi Province, as well as 2 students from Elementary School B, 1 student from Middle School A, and 1 student from Middle School B. In this study, we aimed to characterize the incidence pattern of individuals clinically suspected of having varicella, determine the extent of exposure, and trace the likely index cases of infection.

These students from other schools were included because they shared the same time slots and physical spaces with the index-class students—either at a private after-school academy or within a household setting—during the exposure window (22 May–5 June 2023) and developed clinically compatible varicella within the maximum incubation period of 21 days after that contact.

During the observation period (22 May–26 June 2023), 23 of the 89 school members developed a vesicular or maculopapular rash that met the clinical case definition of varicella and were interviewed by telephone (or through their legal guardians) to obtain detailed symptom histories. The remaining 75 school contacts, who had not yet developed compatible symptoms, were followed prospectively by passive surveillance based on daily teacher reports and a review of absenteeism logs.

### 2.4. Data and Sample Collection

Telephone interviews were conducted with the study participants using the “Suspected Varicella Patient Epidemiological Investigation Form”. An onsite epidemiological investigation was carried out at the school on 7 June. These interviews were conducted primarily with the patients’ legal guardians and, when necessary, directly with pediatric patients who were capable of effective communication. The form covered 11 domains: institution information; patient demographics; school or occupational affiliation; high-risk conditions; clinical signs and course; health-care utilization during the three weeks preceding rash onset; laboratory testing; previous varicella history; vaccination history; source-of-infection investigation (21-day international-travel and symptomatic-contact history); and contact tracing.

On 9 June 2023, oropharyngeal swab specimens were collected from 2 of the 23 affected individuals who had consented to testing and subsequently submitted to the Gyeonggi Institute of Health and Environment for polymerase chain reaction (PCR) testing.

### 2.5. Investigation Procedure

On 7 June 2023 (Wednesday), the public health authorities informed both the facility and Gyeonggi Province about the ongoing varicella outbreak and dispatched epidemiological investigators and infectious disease control personnel to conduct onsite investigations.

Additional inquiries were conducted by school health teachers to identify common exposure sources other than the classroom environment. Individual exposure was assessed through a case-by-case epidemiological investigation.

Each health teacher asked the affected students about (1) classroom seating arrangements and the frequency of contact with other cases; (2) shared activities such as lunch, after-school clubs, school-bus use, or extracurricular lessons; (3) compliance with mask wearing and hand hygiene; and (4) participation in social gatherings during the incubation period [20]. These data were integrated into a case-by-case epidemiological assessment of individual exposure.

Telephone interviews were conducted with the affected individuals, their parents, and facility managers.

Following the field investigation, the school health teacher was instructed to identify close contacts—collecting information on vaccination status, recent international travel, and the presence of any unvaccinated foreign-born students—and to disinfect and ventilate all areas of the facility regularly. Recommendations were provided to encourage varicella vaccination, monitor individuals displaying varicella-like symptoms throughout the outbreak, and seek medical evaluation if suspicious symptoms arose. Teachers and students were advised to maintain proper hand hygiene and to continue routine environmental disinfection [2,20].

For clinically diagnosed cases, the standard epidemiological investigation form was administered either to the child (if communicative and ≥7 years old) or to a legal guardian. When necessary, interviews with close contacts were undertaken to document the timing and intensity of exposure to the index patient, involvement in extracurricular or social activities, use of shared transportation, adherence to mask wearing, and any recent varicella-like symptoms. Testing was recommended in all 23 identified individuals.

As soon as the outbreak was confirmed, administrators of the school and its affiliated private academies were contacted to verify patient contact frequency and facility conditions and were provided with infection-control guidance; they were instructed to refer any individuals with suspected varicella to medical care without delay. Staff were also trained in passive surveillance for additional cases and their contacts, and daily reporting with bidirectional information sharing was maintained until the outbreak was officially declared over.

### 2.6. Statistical Analysis

Outbreak characteristics were summarized with descriptive statistics (frequencies, proportions, means, and standard deviations) calculated in Microsoft Excel 2016. Epidemic curves and transmission-network diagrams were generated to illustrate temporal and spatial patterns, while relationship diagrams were created in Microsoft PowerPoint 2016 to visualize links among cases.

### 2.7. Ethics

This study complied with the regulations of the Korean Disease Control and Prevention Agency, Ministry of Health and Welfare, and local governments in Gyeonggi Province, following the Bioethics and Safety Act (Article 15) and Infectious Disease Control and Prevention Act (Article 18). Therefore, additional ethical approval from an Institutional Review Board (IRB) or Ethics Committee was not required. Individual written consent is not required when information is obtained in the course of a compulsory public-health investigation conducted under the above Act. All personal information was removed before data analysis. Despite these legal regulations in Republic of Korea, if students’ parents refused epidemiological investigations such as blood tests or interviews, they were excluded from the investigation.

## 3. Results

### 3.1. General and Clinical Characteristics

An epidemiological investigation was conducted among the 23 identified individuals. Of these, 2 individuals were confirmed through varicella diagnostic tests, whereas 21 were diagnosed as individuals suspected of having varicella by medical institutions based on clinical symptoms without confirmatory tests. None of the 23 patients belonged to high-risk groups such as immunocompromised individuals (including patients with immunodeficiency disorders and cancer, as well as immunosuppressed individuals) or unvaccinated persons (Table 1). No student or teacher reported international travel within the 21-day pre-rash period, and no foreign-born students lacking varicella vaccination were identified.

When compiling symptoms for the 23 patients (including overlaps), all exhibited rash symptoms (100%), 6 reported fevers, and 3 reported other symptoms (headache, muscle pain, and cough). The mean duration from symptom onset to diagnosis was 1.7 days. All patients fully recovered after outpatient treatment without any complications or history of hospitalization (Table 2).

### 3.2. Epidemiological Description of the Outbreak

*Vaccination background:* According to the national immunization registry, all 85 students at OO Elementary School had received a first dose of the varicella vaccine between 12 and 15 months of age, yielding 100% coverage. Only one pupil had obtained a second dose, and vaccination histories for four teachers could not be verified—three were unsure of their status, whereas one reported a previous episode of varicella.

*School exposure:* Two index cases were clinically diagnosed with varicella on 6 June 2023 in Grade 5, Class 6. Active surveillance of 83 classroom or shared-space contacts from 4 to 10 June identified 12 additional cases, corresponding to a contact-specific attack rate of 14.5% (12/83); including the two index students, the overall school attack rate was 16.5% (14/85).

*Household spread:* The 23 epidemiologically linked school cases generated 58 household contacts, 6 of whom developed secondary infection, giving a secondary household attack rate of 10.3%. No tertiary household cases were detected during the observation period. This finding indicates that transmission was effectively interrupted after the secondary generation, most likely because secondary patients were promptly isolated and their household contacts were actively monitored.

*Exposure to private after-school academies:* Field investigation identified several private after-school academies attended by the students, of which Academy A—a mixed-grade physical-education institute—held daily sessions for approximately 80 children. Observations showed 50–60 students from multiple schools gathering simultaneously between 14:00 and 18:00. Passive surveillance subsequently identified five secondary cases epidemiologically linked to Academy A, two of which were transmitted within households, underscoring the role of this venue as a transmission bridge across otherwise unconnected families. Because complete enrolment lists were unavailable, an academy-specific denominator could not be established, and the associated attack rate is therefore described qualitatively.

*Participation in private after-school institutes:* Of the 23 school-associated cases, 15 (65.2%) reported attending at least one private academy during the likely exposure window, and 11 of these students specifically frequented Academy A. The remaining 8 cases (34.8%) stated that they had not participated in any extracurricular lessons (Table 3).

**Figure 1:**
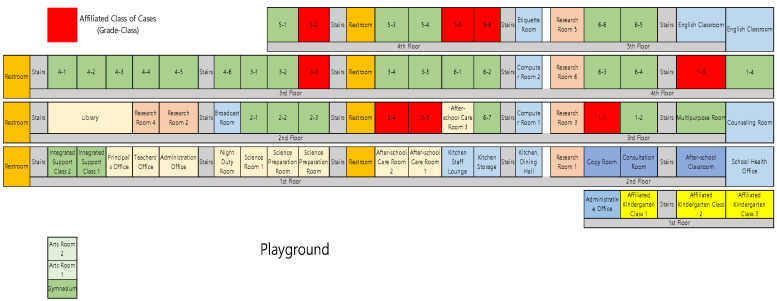
Floor plan of Elementary School A classrooms.

*Spatial–environmental factors inside the school:* Elementary School A is L-shaped, with a four-storey left wing and a five-storey right wing connected by a shared corridor (Figure 1). The initial classroom (Grade 5-6) and two additional affected Grade 5 classes (5-2 and 5-5) are situated on the fourth floor, where they share a restroom and twin drinking fountains. Further cases appeared on the third-floor left wing (Grades 3-3 and 1-3, which use separate facilities) and on the second-floor right wing (Grades 2-4 and 2-5, which share facilities); a kindergarten on the right-wing first floor reported no cases.

*Behavioral context:* After national COVID-19 mask mandates were lifted, students generally did not wear masks, classroom ventilation was inconsistent, and lunch was eaten inside classrooms. The school was closed from 3 to 6 June for a weekend break, a discretionary closure day, and Memorial Day, and later observed a summer vacation from 25 July to 24 August.

**Figure 2:**
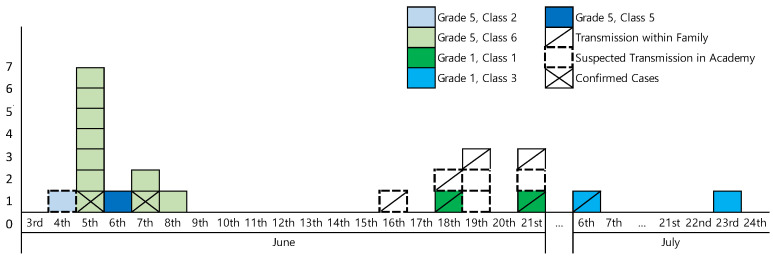
Epidemic curve of a varicella outbreak associated with Elementary School A.

*Temporal pattern:* Symptom-onset data showed two distinct peaks: twelve cases occurred between 4 and 8 June, and eleven cases emerged from 16 June to 23 July, producing a multimodal curve consistent with serial, person-to-person transmission (Figure 2). No additional school- or academy-associated cases were reported after 14 June, and the predefined 42-day surveillance window elapsed without further infections, fulfilling national criteria for declaring the outbreak over.

### 3.3. Laboratory Test Results

With parental consent, oropharyngeal swabs were collected from 2 of the 23 affected students on the day after the outbreak was recognized, because all participants were minors. The specimens were forwarded to the Gyeonggi Provincial Institute of Health and Environment for PCR targeting varicella-zoster virus, and both tests returned positive results.

Under the KDCA guidelines, an educational facility must initiate an outbreak investigation when two or more suspected cases occur within a three-week interval. The same guidelines stipulate that laboratory confirmation is required in at least two patients or 10% of all suspected cases, whichever number is greater; by testing two students, our investigation satisfied this criterion.

Further molecular work, such as sequence typing, was not feasible. Additional specimens could be obtained only with parental consent, and most parents and students declined, limiting the scope of laboratory analyses.

### 3.4. Estimation of Infection Source

(1) Incubation and risk-exposure periods

Although the exact source of infection for the first case is unknown, a review of school-attendance records and symptom-onset dates suggests that the first wave of cases (Grade 5 students) was exposed in the same classroom during the prodromal phase (22–23 May 2023) of the index patient’s twin sibling. Subsequent cases linked to academies and households constituted a second wave, and the mean interval between the two waves was 13 days, which falls within the recognized incubation period for varicella.

(2) Causative Pathogen

All 23 patients were clinically diagnosed with suspected varicella and showed symptoms such as fever and rash. Two students tested positive for varicella-zoster virus by PCR, confirming that the outbreak was caused by *Human alphaherpesvirus 3*. The average interval from the presumed source patient (the twin sibling) to onset of symptoms in first- and second-wave cases was 13 days, aligning with the 10–21-day incubation period of varicella.

**Figure 3:**
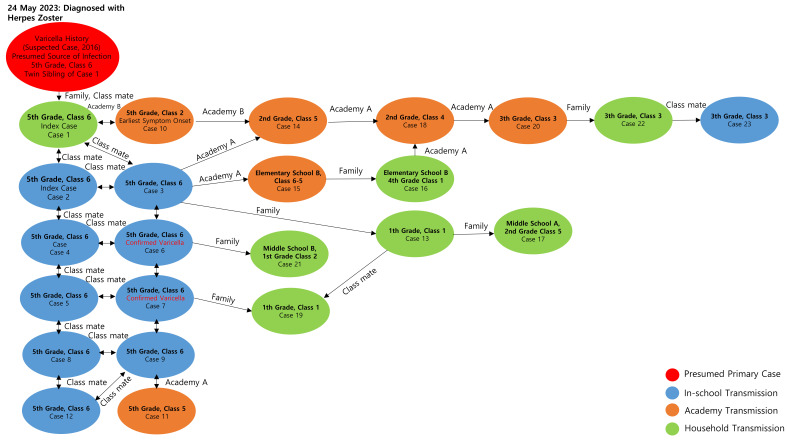
Network diagram of varicella transmission associated with Elementary School A outbreak

(3) Presumed Index Patient (Table 4, Figure 3)

- Family of Case 1

This instance involved a twin sibling of the first affected individual, who also belonged to Class 6 of the fifth grade. The individual developed a rash on 24 May and was diagnosed with herpes zoster at a dermatology clinic. Records from the Integrated Disease and Health Management System confirmed a case of suspected varicella infection in 2016. As this individual exhibited a rash and was diagnosed with herpes zoster but was not subject to school attendance restrictions or isolation, and given that numerous varicella cases, including among family members, occurred in Class 6 after the varicella incubation period (10 days), this individual is presumed to be the index patient. Although varicella arising after contact with herpes zoster is rare, it has been recognized since the 1960s [21] and continues to be reported [22,23]. The patient reported no travel history in the past 3 weeks or contact with patients with varicella or disseminated herpes zoster, making it impossible to determine the initial source of infection for the index patient.

b. Cases 1 and 10

Case 1 has traditionally been labeled the index patient; however, several classmates developed symptoms between 1 and 3 June, which is less than the minimum 10-day incubation period after the twin sibling’s rash on 24 May. Case 10 exhibited the earliest symptom onset within the cluster, yet this date (4 June) was essentially simultaneous with other early cases, making it difficult to designate Case 10 as the primary source. Consequently, the twin sibling of Case 1 remains the most credible index patient.

## 4. Discussion

This investigation documented a 23-case varicella outbreak that began with two fifth-grade index cases and spread through households and private after-school academies, even though first-dose coverage exceeded 90%. Although no formal statistical modeling was undertaken, the absence of additional school- or academy-associated cases for two maximum incubation periods (42 days) after control measures were implemented suggests that the integrated response helped interrupt transmission. This study was limited by sparse laboratory confirmation—polymerase-chain-reaction positivity in only two patients—and by incomplete attendance records from the academies.

One- and two-dose varicella vaccination schedules substantially reduce disease incidence but do not confer sterilizing immunity. In a large Chinese study, breakthrough attack rates were 0.4% after a single dose and 0.1% after two doses, corresponding to vaccine-effectiveness estimates of 85% and 96%, respectively [7]. Our findings therefore support the possibility that breakthrough clusters can still emerge in highly vaccinated settings when close contact is intense.

*What is new:* Previous Korean reports have focused almost exclusively on in-school transmission; our investigation indicates that private academies—attended by >80% of primary-school children—functioned as amplifiers of transmission across grade levels, classrooms, and even different schools, rather than being the primary cause of the outbreak itself. Because varicella vaccination (one-dose coverage ≥ 95%) does not confer sterilizing immunity [7], breakthrough cases can still arise; intense, inter-school mixing in academies simply facilitated their wider dissemination. Consequently, targeted infection-control measures in these venues were essential to halt further spread.

*Why does this matter globally:* Wide heterogeneity exists in national policies. Among the 30 EU/EEA member states, first-dose varicella vaccination falls into four categories: mandatory and fully publicly funded in three countries (Italy, Latvia, and Hungary); recommended and offered free of charge in twelve (including Germany); recommended but paid out of pocket in four (Austria, Belgium, Cyprus, and the Czech Republic); and not yet introduced in fifteen (including Ireland); where varicella vaccination is recommended but not reimbursed, uptake is exceedingly low—Belgium reports <2.5% two-dose coverage among toddlers, illustrating that funding policy can be as decisive as clinical recommendations in achieving population-level protection [5,6]. In Ireland and the other fourteen non-adopting countries, low coverage means many children acquire immunity through natural infection [4,5,24]. In many low- and middle-income countries, routine varicella immunization has yet to be introduced at all [9]. Our investigation demonstrates that even where coverage is high, pockets of un- or under-vaccinated individuals, together with waning immunity, can ignite outbreaks; systematic field investigations therefore remain essential to refine dosing schedules, evaluate vaccine performance, and advocate for program introduction or expansion. Because breakthrough infections and their complications continue to be reported even in highly vaccinated countries, outbreak investigations are all the more important.

*Epidemiologic context in Korea*: National surveillance shows varicella notifications rebounding toward pre-COVID-19 levels during 2023, mirroring international trends of resurging vaccine-preventable diseases after the relaxation of distancing measures [23]. The present cluster illustrates how renewed social mixing, especially in extracurricular settings, can accelerate transmission. In addition, the Republic of Korea still follows a single-dose varicella schedule, creating cohorts whose immunity may wane over time [3]. In a nationwide Korean birth-cohort study, hazard-ratio-based vaccine effectiveness (VE) declined from 86% during the first year after vaccination to 49.9% six years later [8]. By contrast, a cohort analysis from Shanghai Pudong reported that two-dose risk-ratio-based VE remained at a mean of 96% (95% CI 93–99%) [7]. These findings suggest that waning immunity among students who received only a single dose five to thirteen years earlier could have facilitated the present outbreak.

*Study limitations:* First, parental refusal limited the number of PCR tests performed and therefore prevented sequencing. Second, incomplete rosters from the after-school academies hindered the calculation of setting-specific attack rates and likely resulted in under-ascertainment.

*Future directions:* Because breakthrough infections and their associated complications continue to be reported even in settings with high vaccination coverage, outbreak investigations are becoming increasingly important. Systematic investigations of outbreaks in extracurricular settings are needed to refine dosing intervals, assess long-term vaccine effectiveness, and guide targeted infection-control policies. In parallel, longitudinal serologic studies in both high- and low-coverage countries are required to clarify the role of waning immunity and to inform decisions on booster doses. Our findings do not negate the clinical benefits of Korea’s single-dose program; breakthrough cases were generally mild, indicating that a single dose still provides substantial protection [7]. However, lower long-term seroconversion rates and recurrent outbreaks in highly vaccinated cohorts suggest that adding a routine second dose could further reduce transmission. Therefore, we support initiating a national policy dialogue—guided by evidence from countries that use two-dose schedules [3] and by a formal cost-effectiveness evaluation tailored to Korea—to determine whether a second dose should be incorporated into the national immunization program.

## 5. Conclusions

Breakthrough varicella clusters can still arise in settings where one-dose coverage exceeds 90%, especially when intense mixing occurs in extracurricular venues. Although incomplete patient sampling and missing attendance data limited molecular confirmation and precise attack-rate estimates, the investigation identified private academies as critical yet under-regulated transmission nodes.

Implementing coordinated infection-control measures simultaneously in the school and its affiliated private academies rapidly curtailed transmission, highlighting the value of an integrated response across all educational settings. Closer collaboration between public-health authorities and private educational institutions will enable more comprehensive data collection and faster diagnostic testing, thereby improving the efficiency of future outbreak investigations and control efforts.

## Figures and Tables

**Table 1:** General characteristics of varicella cases associated with the Elementary School A outbreak (N = 23).

Characteristic	Category	Frequency (N)	Percentage (%)	Mean ± SD
Gender	Male	14	60.9	
	Female	9	39.1	
Age (years)	6	1	4.3	
	7	4	17.4	
	8	2	8.7	
	9	1	4.3	
	10	5	21.7	9.78 ± 2.04
	11	7	30.4	
	12	1	4.3	
	13	1	4.3	
	14	1	4.3	
Affiliation	Elementary School A	19	82.6	
	Elementary School B	2	8.7	
	Middle School A	1	4.3	
	Middle School B	1	4.3	
Extracurricular Activity	None	8	35.0	
	After-school Programs	2	9.0	
	Private Academies	15	65.0	
Vaccination Status	1 Dose	22	95.7	
	2 Doses	1	4.3	
Case Classification	Confirmed Case	2	8.7	
	Suspected Case	21	91.3	

**Table 2:** Clinical characteristics of varicella cases associated with the Elementary School A outbreak (N = 23).

Characteristic	Value
Symptoms, Number of Patients (%) *	
Rash	23 (100.0)
Fever	6 (26.1)
Headache	1 (4.3)
Myalgia	1 (4.3)
Cough	1 (4.3)
Initial Rash Location, Number of Patients (%) *	
Trunk	17 (78.3)
Face/Neck	5 (21.7)
Limbs	1 (4.3)
Number of Vesicular Lesions, Number of Patients (%)	
<50	16 (69.6)
50–249	7 (30.4)
Time from Symptom Onset to Report (Days)	
Median	2
Range	0–5
0 days	2 (8.7)
1 day	9 (39.1)
2 days	8 (34.8)
3 days	2 (8.7)
4 days	1 (4.3)
5 days	1 (4.3)

* Multiple responses allowed.

**Table 3:** Distribution of 23 varicella cases by private academy and after-school registrations.

Academy/Activity	Patients, N (%) *	Registrations, *n* (%) ^†^
Academy A	6 (26.1)	6 (22.2)
Academy B	5 (21.7)	5 (18.5)
Academy C	3 (13.0)	3 (11.1)
Academy D	2 (8.7)	2 (7.4)
Academy E	2 (8.7)	2 (7.4)
Academy F	2 (8.7)	2 (7.4)
Academy G	2 (8.7)	2 (7.4)
Academy H	1 (4.3)	1 (3.7)
Academy I	1 (4.3)	1 (3.7)
Academy J	1 (4.3)	1 (3.7)
After-school (in-school) class	2 (8.7)	2 (7.4)
No academy attended	8 (34.8)	—
Totals	23 (100.0)	27 (100.0)

* The Patients column uses N = 23 as its denominator, whereas the Registrations column uses *n* = 27. ^†^ Because a single child may register for more than one academy, the total number of registrations (*n* = 27) exceeds the number of patients (N = 23).

**Table 4:** Affiliation and academy details of varicella outbreak cases associated with Elementary School A.

Serial No.	Report Date	Affiliation(Grade-Class)	Academy ^†^
1	6 June	5-6	B, E, I
2	6 June	5-6	D
3	7 June	5-6	A
4	7 June	5-6	-
5	7 June	5-6	-
6	7 June	5-6	F
7	8 June	5-6	H, J
8	8 June	5-6	-
9	8 June	5-6	B, C
10	9 June	5-2	B, E
11	10 June	5-5	C, After-school Class
12	10 June	5-6	D
13	19 June	1-1/ Case 3 Family	-
14	19 June	2-5	A, B, G
15	19 June	Elementary School B 6-5	A, B
16	19 June	Elementary School B 4-1/ Case 15 Family	A
17	20 June	Middle School A 2-5/ Case 3, 13 Family	-
18	20 June	2-4	A, F, G
19	21 June	1-1/ Case 7 Family	-
20	23 June	3-3	A, After-school Class
21	23 June	Middle School B 1-2/ Case 6 Family	-
22	7 July	1-3/ Case 20 Family	-
23	24 July	1-3	C

^† ^ Letters (A–J) denote anonymized academy identifiers used for confidentiality.

## Data Availability

Data available on request due to privacy.
